# A Higher Adherence to the ALINFA Nutritional Intervention Is Effective for Improving Dietary Patterns in Children

**DOI:** 10.3390/children11050559

**Published:** 2024-05-07

**Authors:** Natalia Vázquez-Bolea, Naroa Andueza, Marta Cuervo, Santiago Navas-Carretero

**Affiliations:** 1 Department of Nutrition, Food Sciences and Physiology, Faculty of Pharmacy and Nutrition, University of Navarra, 31008 Pamplona, Spain; nvazquezb@unav.es (N.V.-B.); nandueza@alumni.unav.es (N.A.); snavas@unav.es (S.N.-C.); 2 Center for Nutrition Research, University of Navarra, 31008 Pamplona, Spain; 3 Navarra Institute for Health Research (IdiSNA), 31008 Pamplona, Spain; 4 Biomedical Research Networking Center for Physiopathology of Obesity and Nutrition (CIBERObn), Institute of Health Carlos III, 28029 Madrid, Spain

**Keywords:** dietary habits, Mediterranean Diet, child nutrition science, guideline adherence, health education, lifestyle, dietary modifications

## Abstract

Food patterns are deteriorating and, consequently, not meeting nutritional recommendations. Learning about the adherence to a diet is crucial for understanding children’s dietary habits. The objective of the present analysis was to assess the degree of compliance with the ALINFA nutritional intervention and the effectiveness of adherence groups, and to evaluate potential baseline factors predicting a higher adherence to the intervention. A total of 44 children aged 6 to 12 years-old participated in the eight-week intervention. A two-week dietary plan was specifically designed, providing participants with food products, ready-to-eat dishes, and recipes. An intake of 75% of calories of the prescribed diet was defined to divide the participants into high- and low-adherence groups (HA/LA, respectively). From the 44 participants, 24 showed a LA to the intervention, whereas 20 of them were in the HA group. Diet quality improved in both groups (*p* < 0.001), mainly by increasing cereals and nuts, and reducing pastries. A decrease in BMI z-score was observed (LA: *p* < 0.001; HA: *p* = 0.021). Fat mass (*p* = 0.002), LDL-c (*p* = 0.036), and CRP (*p* = 0.023) reductions were only achieved in the HA group, whereas leptin decreased only in the LA group (*p* = 0.046). All participants ameliorated their dietary habits, but those with better diet quality at baseline experienced greater enhancements in their nutritional status.

## 1. Introduction

As a result of globalization, food patterns are being affected, to the detriment of nutritional quality [1]. What led society to this situation is not straightforward, but fortunately it is manageable, and dietary habits in infancy play a key role [2]. Children are a vulnerable population, and the establishment of healthy habits through lifestyle interventions during the early years of life can prevent numerous issues in adulthood [3]. However, the environment does not help achieve a healthy diet [4]. Ultra-processed foods [UPFs] are available in stores, fast-food restaurants are proliferating rapidly, and portion sizes are tending to increase, increasing the daily intake [4,5]. Food marketing does not promote the creation of healthy habits in children [6]. Indeed, overweight and obesity rates are increasing. The most recent data from the ALADINO study in Spain in 2019 indicated that 23.3% of schoolchildren had overweight and 17.3% had obesity [7]. 

Children represent a primary target for food and health interventions, as nutritional behaviors acquired when young survive into adulthood [8]. Children in North America, Europe, and Oceania do not achieve the recommendations for fruits, vegetables, and legumes, whereas they surpass the recommended salt intake [9]. For every five calories ingested, one comes from junk food [10]. The ALADINO study showed that 76.2% of children needed to improve their diet quality, according to the score of the Mediterranean Diet Quality Index for children and adolescents (KIDMED) [7]. In this context, Spanish children’s eating habits are worsening, and urgent action needs to be taken [11]. Implementing strategies that provide education about nutrition in the initial stages of life can prevent numerous public health issues [12]. However, food choices and eating behaviors are affected by countless factors, including innates ones such as genetics, sex, and age, but also environmental ones: parents and family, socioeconomic status, demographics, and own children’s food preferences [13]. Parents and home environment play a pivotal role in the development of dietary habits. They are a critical pillar, and parents thus need to be conscious about their acts because children will emulate their caregivers. Parents have to learn about nutrition and participate in the development of their children’s dietary habits [14]. However, schools also play a major role. Education about nutrition at primary schools appears to be a positive influence for making healthier food choices [15], as the lack of nutritional knowledge seems to be determinant in food attitudes and behaviors [16]. Indeed, according to a study performed in European countries, both children and elementary and primary school teachers in Spain have the lowest nutritional knowledge [17]. Several organizations have created visual guides and specific materials to promote healthy eating among children; for example, the Harvard T.H Chan School of Public Health created the Healthy Eating Plate [18]. In Spain, the Spanish Society for Community Nutrition created a food pyramid to offer nutritional recommendations for the youth population. In addition, to maintain a healthy lifestyle, 60 min of physical activity is recommended every day [19,20]. 

In that sense, performing adequate dietary interventions in children to increase and improve their dietary habits could be of great interest, but these interventions to be fulfilled. Nutritional interventions based on the Mediterranean Diet [MD] in healthy children have not been found, but studies show that in healthy adults, the MD is essential for improving health [21]. Indeed, for children and adults with non-communicable diseases such as obesity, the MD is crucial for achieving good health [22,23,24,25]. A factor that must be considered is the adherence to the intervention, which is necessary for the compliance of studies. Few studies have been published about adherence, and none of these have been conducted in children. In adults, the literature shows that the more participants adhere to a diet or an intervention, the more benefits they will gain [26,27]. To optimize the success of interventions in children, certain factors need to be considered. Face-to-face sessions must be applied by experts on the topic, the duration of the intervention must be considered, and some activities based on age need to be used [28]. In addition, school- and community-based interventions also appear to be promising for decreasing the risk of developing diseases and for the promotion of consumption of healthy food [29,30]. Active approaches, such as performing cooking or gardening seminars, or giving children free healthy foods, seem to be beneficial both for future health outcomes and for nutritional education [31]. 

Effective nutritional interventions in children are imperative as habits acquired in childhood will persist throughout the entire life cycle [8]. In this sense, a project for the development of healthy dietary habits in childhood, named ALINFA, was implemented. It aimed to generate knowledge and to improve the provision of healthy food for children aged 6 to 12 years, by providing participants with healthy foods and recipes for home meals. In this sense, the objective of this research was to study the degree of compliance with the nutritional intervention and its effectiveness between adherence groups, in children belonging to the intervention group of the ALINFA study. We sought to evaluate the differences in anthropometric, body composition, and biochemical measurements, and lifestyle habits, after the intervention, by adherence group, as well as to identify baseline factors that may help predict a higher adherence to an intervention.

## 2. Materials and Methods

An eight-week parallel, randomized controlled trial was performed. The Center for Nutrition Research at the University of Navarra was in charge of its development. The intervention was performed during 2021 and was designed following the Good Clinical Practice stated by the Declaration of Helsinki [32]. Ethical approval was obtained from the Research Ethics Committee of the University of Navarra (ref. 2021.027). The study was registered in the ClinicalTrials.gov database (NCT05249166).

Children between the ages of 6 and 12 were invited to participate in the study, provided they met specific criteria. They had to have lunch at home or could bring their own food to the school canteen; have an adequate cultural level for the understanding of the study; and provide agreement to participate voluntarily. Exclusion criteria encompassed the presence of relevant functional or structural abnormalities, uncontrolled endocrine disorders, any type of cancer, a weight loss of 3 kg or more in the last 3 months, or allergy to any component of the products under study.

### 2.1. Description of the Intervention

During the eight weeks of intervention, the following visits were scheduled: information and screening visit (V0), initial visit (V1), follow-up visit (V2), and final visit (V3). Participants and parents willing to participate signed the informed consent form, where all the necessary information regarding the study was explained. They were randomly allocated to the intervention group or the control group, following a 2:1 ratio. Siblings were allocated in the same group to facilitate compliance with the intervention; the procedure was to randomly assign the older sibling. During V1, body composition measurements were taken from children, as well as blood samples. Parents filled in the dietary and lifestyle questionnaires. The children allocated to the ALINFA group also received tailored products, recipes, and instructions on preparation. The follow-up visit for the intervention group was face-to-face as it was necessary to provide the participants with food for the second part of the study. In relation to V3, the procedures were the same as those for V1.

The intervention group followed a healthy dietary plan. A two-week menu was planned, and repeated four times throughout the intervention (Supplementary Materials Table S1). Two different recommendations regarding portion sizes were made according to age. Participants from 6 to 9 years old were advised to consume smaller portions than participants from 10 to 12 years old. The two-week menu had a total energy intake of 1691 for the younger group, and 1855 kilocalories for the older group. The proposed menu was designed following nutritional recommendations, in compliance with the Acceptable Macronutrient Distribution Ranges [33]. No energy restriction was imposed on any of the menus. The menu given to the ALINFA group was based on a healthy diet that included all food groups. It was a fixed full-day meal plan. Five meals were planned for each day. Food products, ready-to-eat dishes, and healthy recipes, which were specifically designed for this intervention by food companies and research centers, were provided to participants (Supplementary Materials Table S2). For this, several entities took part: The National Centre for Technology and Food Safety (CNTA), Public University of Navarra (UPNA) and University of Navarra (UNAV), Grupo Apex (Aperitivos y Extrusionados, S.A.), Harivenasa S.L., Alimentos Sanygran S.L., Industrias Alimentarias de Navarra (IAN) S.A.U. and Irigoyen Comedor Saludable S.L. Nutritional information regarding the ALINFA menu can be seen in Supplementary Materials Table S3.

### 2.2. Study Measures

At V1 and V3, anthropometric, body composition, and biochemistry measurements were taken to look for differences after the eight-week nutritional intervention, while parents or caregivers filled out dietary and lifestyle questionnaires. 

#### 2.2.1. Anthropometry and Body Composition

The following measurements were conducted under fasting conditions at V1 and V3. For height (m), a wall stadiometer was used (Seca 220, Vogel and Halke, Hamburg, Germany). A bioimpedance weighting scale (SC-330, Tanita, Tokyo, Japan) was used to assess weight (kg) and to estimate fat, lean, and muscular mass in kilograms (kg). Body mass index (BMI) was calculated using the formula weight (kg)/height (m)^2^, and BMI z-score was also interpreted following the WHO classification [34]. Waist circumference was measured using a flexible and inelastic tape. Systolic and diastolic blood pressure was measured with an automatic sphygmomanometer (IntelliSense. M6, OMRON Healthcare, Hoofddorp, The Netherlands). 

#### 2.2.2. Biochemical Measurements

Blood extraction was performed at V1 and V3, under fasting conditions if children and caregivers provided consent. Samples were processed in a standard centrifuge (Eppendorf 5804R, Hamburg, Germany) to obtain serum and plasma. A Pentra C200 autoanalyzer (Horiba ABX Diagnostics, Montpellier, France) was used to measure the serum glucose (mg/dL), total cholesterol (TC; mg/dL), and high-density lipoprotein cholesterol (HDL-c; mg/dL), by colorimetric methods. An automated ELISA processing system DSX^®^ Dynex Technologies (Palex, Chantilly, VA, USA) was utilized to measure insulin (μIU/mL), alpha tumor necrosis factor (TNF-α; pg/mL), leptin (ng/mL), and C-reactive protein (CRP; mg/dL). The Friedewald formula [35] was used to quantify the low-density lipoprotein cholesterol (LDL-c; mg/dL) concentration. HOMA-IR values were also calculated [36].

#### 2.2.3. Lifestyle Habits Assessment

Caregivers were asked to fill in a series of questionnaires to assess their lifestyle. The quality of children’s diet and adherence to the Mediterranean Dietary Pattern was assessed through the KIDMED questionnaire [37]. This consists of 16 items with a dichotomic answer of “yes” or “no”. Items include takes a piece of fruit or fruit juice every day, has a second piece of fruit every day, has fresh or cooked vegetables regularly once a day, has fresh or cooked vegetables more than once a day, consumes fish regularly, goes more than once per week to a fast-food restaurant, likes pulses and eats them more than once per week, consumes pasta or rice almost every day, has cereals or grains for breakfast, consumes nuts regularly, uses olive oil at home, skips breakfast, has a dairy product for breakfast, has commercially baked goods or pastries for breakfast, takes two yoghurts and/or some cheese daily, and takes sweets and candy several times every day. The KIDMED index score can range from minus 4 to 12 points. Depending on the total score, adherence to the MD and diet quality can be classified into 3 different categories: low diet quality (less than 3 points), need to improve diet quality (4 to 7 points), and optimal MD quality (more than 8 points). The KINDL questionnaire (Child Quality of Life Questionnaire) was used to assess the quality of life of the children [38,39,40]. This validated questionnaire consists of 12 items belonging to 6 scales: physical well-being, emotional well-being, self-esteem, family, friends, and everyday functioning (school). An additional scale regarding disease with six items is also included, and only needed to be answered in case the child was hospitalized or ill for a prolonged period. A three-point Likert-type scale is used to evaluate this questionnaire (0 = never; 1 = sometimes; 2 = very often). All values from the scales and sub-scales were transformed into a score ranging up to 100 points, meaning that the higher the score, the higher the perceived quality of life. Physical activity was measured with the validated Physical Activity Questionnaire for Children (PAQ-C) [41,42]. The Child Eating Behavior Questionnaire (CEBQ) was also used to assess the different eating behaviors in children [43,44]. This validated questionnaire consists of 35 questions with a five-point Likert-type scale answer (0 = Never; 1 = Rarely; 2 = Sometimes, 3 = Often, 4 = Always). Five questions are assessed with an inverted scale (4 = Never; 3 = Rarely; 2 = Sometimes, 1 = Often, 0 = Always). The 35 questions are divided into eight different scales to assess the following eating behaviors: food responsiveness, enjoyment of food, emotional overeating, desire to drink, satiety responsiveness, slowness in eating, emotional undereating, and food fussiness.

#### 2.2.4. Dietary Intake Assessment

To measure the dietary intake, participants in the intervention group completed food records. Sheets with the planned menu were handed to participants, where they recorded the proportion of each food they consumed: none, one quarter (¼), half (½), three-quarters (¾), or all of the prescribed diet. To calibrate the dietary intake, NUTRIUM (Healthium-Healthcare Software Solutions, S.A.; https://www.nutrium.com) was used. With the information of the food records, dietary intake calibrations were undertaken for each participant, for all meals and for the eight-week intervention. All extra foods that were not in the planned diet were not initially included in the calibration. Once the food records were analyzed, children were categorized into two groups according to their compliance with the prescribed diets, into low adherence (LA) and high adherence (HA). In this context, participants consuming less than 75% of the prescribed kilocalories depending on age were assigned to the LA group. On the contrary, subjects that consumed more than 75% of the prescribed kilocalories were assigned to the HA group. Afterwards, a second calibration was conducted including all the foods consumed, both those that were on the planned diet and those that were not, to have information on the total energy and dietary intake.

### 2.3. Statistical Analysis

All the analyses were performed in STATA 15.1 (StataCorp LP, College Station, TX, USA). Normal distribution was assessed with the Shapiro–Wilk test. Variables are described as mean and standard deviation if normally distributed, or as median and interquartile range if not normally distributed. The paired Student’s *t*-test or Wilcoxon test were used to identify differences within the same group. To identify differences between groups, the Student’s *t*-test or Mann–Whitney U test was selected. Statistical significance was established when the *p* value was lower than 0.05 for all tests. Categorical data are presented as percentages. To identify differences within groups, the McNemar test was used, while the chi-square test was performed to look for differences between the groups. Repeated measures ANOVA was also utilized to identify differences in energy and macronutrients during the eight-week intervention. When significantly different, the Tukey post hoc test was performed. Linear regression models were performed to identify factors that could predict a higher or lower percentage of adherence to the intervention. It was decided to consider the total score of the KIDMED index and its 16 items as predictive factors. When executing the regressions, intra-cluster correlations between siblings were considered. β-coefficients, with 95% confidence intervals and *p* values, were obtained. Remarkable results from the linear regressions were transformed into graphs constructed in STATA 15.1. 

## 3. Results

For this analysis, 44 subjects in the ALINFA intervention group were included, and subsequently divided into two groups: HA to the intervention (n = 20) and LA to the intervention (n = 24). Table 1 shows the percentage of energy and macronutrients consumed with regard to the prescribed diet. The LA group mean energy intake (as a percentage of the total diet) was 67.32 ± 7.71% of the prescribed kilocalories, and the HA group’s result was 85.02 ± 7.98%. Repeated measures ANOVA was conducted to find differences during the intervention. Only the fat intake (%) was found to be significant (*p* = 0.029) in the HA group. Fat consumption in weeks three and four was found to be lower than that in the following two weeks after conducting the post hoc Tukey test (80.08 ± 12.76 vs. 85.41 ± 7.78).

Anthropometry, body composition, biochemistry, and questionnaires’ scores are shown in Table 2. From the 24 participants in the LA group, 10 were boys and 14 girls, with a mean age of 9.17 years. In HA group, 8 subjects were boys and 12 were girls, with a mean age of 9.10 years. No significant differences were observed in any baseline parameter, except for the KIDMED index. Participants in the HA group began the intervention with a higher quality of diet than participants in the LA group (*p* = 0.001). When evaluating the effect of the intervention, both groups increased height and reduced BMI (height= LA, *p* < 0.001; HA, *p* < 0.001; BMI= LA, *p* = 0.002; HA, *p* = 0.019). BMI z-score also decreased (LA, *p* < 0.001; HA, *p* = 0.021). The LA group showed an increase in diastolic blood pressure (DBP) (*p* = 0.036). Fat mass (kg) decreased in the HA group (*p* = 0.002), while no significant differences were observed in the LA group. Consequently, differences in fat mass changes between LA and HA groups were observed (*p* = 0.030). LDL-c values significantly decreased only in the HA group (*p* = 0.036). Leptin levels significantly decreased in the LA group (*p* = 0.046), and they also showed a trend in the HA group (*p* = 0.055). CRP decreased in the HA group (*p* = 0.023), which indicates a lower inflammatory state. Focusing on the diet quality, the KIDMED index score increased significantly in both groups (*p* < 0.001), which means an overall improvement in their diet. 

Scores for the quality of diet questionnaires are shown in Table 3. There were no baseline differences in any of the items except for number 2: only 29.17% of children in the LA group had at least two pieces of fruit daily, whereas 60% of subjects consumed them in the HA group (*p* = 0.040). In regard to changes after the intervention, the LA group showed a significant improvement in different items. There was an increase in the percentage of subjects who had a second piece of fruit every day (+25%; *p* = 0.014), had fresh or cooked vegetables regularly once a day (+20.83%; *p* = 0.025), had fresh or cooked vegetables more than once a day (+25%; *p* = 0.014), had cereals or grains for breakfast (+20.84%; *p* = 0.025), or consumed nuts regularly (+29.17%; *p* = 0.019). The number of participants in the LA group who decreased the intake of commercially baked goods or pastries for breakfast also diminished (−29.17%; *p* = 0.020). In regard to the HA group, differences can also be noted. The number of participants who increased the intake of nuts rose by 35% (*p* = 0.015). There was also an increase of 15% in subjects who had cereals or grains for breakfast (*p* = 0.045). Participants in the HA group also increased the intake of a second piece of fruit every day from 60% to 85%, although only a trend towards significance was noticed (*p* = 0.083). A reduction of 45% in the HA group was seen in participants who had commercially baked goods or pastries for breakfast (*p* = 0.008). No significant changes were observed for the remaining items in any group. 

CEBQ scores are annotated in Table 4. There were baseline differences between groups in scales relating to slowness in eating and emotional undereating. The LA group ate slower at baseline than the HA group (*p* = 0.042). Concerning the emotional undereating scale, the HA group had a significantly lower score at baseline than the LA group (1.93 ± 0.67 vs. 2.43 ± 0.72, respectively *p* = 0.023), which means that the LA group tended to eat less in negative contexts, such as anger, tiredness, or sadness. No differences were observed in the CEBQ score, nor were changes observed after the intervention in any of its eight scales.

**Figure 1:**
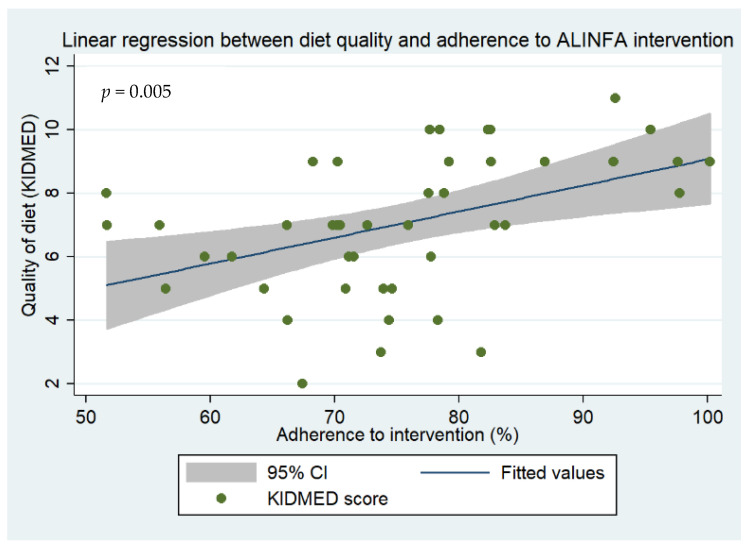
Predictive capacity of quality of diet to the adherence to ALINFA intervention.

After looking at all previous results, the best approach was decided, i.e., to analyze the predictive capacity of different variables on the higher or lower adherence of children to the diet before starting the intervention (Table 5). The quality of diet was found to be significant (Figure 1); the higher the initial diet quality, the greater the adherence to the intervention (*p* = 0.005). Six items from this questionnaire were suggested to be predictive factors. Participants who had a second piece of fruit every day before the intervention had a higher adherence to the diet (*p* = 0.047), improving in 7.12%. Participants who had fresh or cooked vegetables more than once a day before the intervention showed a higher adherence (*p* = 0.007) in 10.38%. Subjects who consumed fish regularly also showed a better adherence to the diet (*p* = 0.001), in almost 10% (β = 9.52). Children who had a dairy product for breakfast before the intervention appeared to better adhere to it (*p* < 0.001; β = 19.55). It was also seen that participants who took two yogurts and/or some cheese daily adhered better to the diet (β = 5.76; *p* = 0.003). On the other hand, participants who took sweets and candy several times every day prior to the intervention had a lower adherence to the diet (*p* < 0.001) in more than 12% (β = −12.50).

## 4. Discussion

Globally, the intervention was effective for all the participants belonging to the intervention group, even if they had HA or LA to the diet, although some extra benefits were seen in the HA group that will be discussed. All this underscores the importance of efficient nutritional interventions in children to establish dietary habits that will endure through the life cycle.

Energy and macronutrient consumption remained stable throughout the study period, proving that the diet was followed without issues, for most participants. This shows that children fulfilled the intervention and did not abandon it. Distributing the free healthy foods provided by the companies appeared to help participants follow an intervention [29]. Only the percentage of fat intake changed during the 8 weeks in the HA group. However, when converted to other measurements, the difference was 3.2 g and 28.8 kilocalories, which makes it physiologically irrelevant [45]. 

As mentioned, height increased in both groups while weight did not change, which inevitably caused BMI to decrease, although the size of the change was minimal. This is in line with other nutritional interventions performed in children [46,47]. However, research on school- and community-based interventions showed that, although the quality of diet improved, BMI did not decrease [48]. All this can be explained in physiological terms, as it is a growth period for children of this age, where changes in body composition occur. Furthermore, another study supports the result of BMI z-score reduction [47]. The change in this parameter in our participants is not concerning as scores between minus two standard deviations and plus one standard deviation are interpreted as normal weight [49]. The reduction in fat mass, and not muscular mass, was observed only in the HA group. This may be due to a higher compliance with the intervention and improved quality of diet, showing the effectiveness of the ALINFA strategy. Other interventions performed in healthy children have also resulted in a decrease in fat mass [50]. All these measurements are valuable and necessary for assessing anthropometric data [51].

Regarding biochemical parameters, the HA group from the ALINFA study exhibited a reduction in LDL-c, consistent with findings in other interventions [52]. In addition, although data from healthy children are lacking, studies performed in children with diabetes and obesity showed that the MD can decrease LDL-c levels, mirroring the observations in our study [23,53]. Given that LDL-c is a cardiovascular risk factor [54], it reinforces the idea of the importance of HA to the MD. A suggested 10% decrease in leptin was observed in the LA group, whereas the HA group did not reach significance; this is possibly attributable to the small sample size, as not all the participants agreed to take blood samples, but a trend towards significance was seen [*p* = 0.055]. These findings contrast with previous data, which indicate that leptin increases during pubertal growth, particularly in girls [55,56,57]. However, this decrease can be explained as leptin is related to weight and BMI, i.e., leptin increases with greater weight and BMI [58,59]. As mentioned, BMI decreased, which can explain the lower leptin. CRP decreased only in the HA group. This could be attributed to the fact that a higher adherence to the MD has been associated with lower levels of inflammation in Spanish adults and adolescents [60,61]. An intake of fruit, vegetables, or fiber is also associated with lower levels of CRP in girls [62], whereas a lower quality of the diet with consumption of UPF is linked to higher CRP serum levels in both infants and adults [63]. In order to establish a connection between both parameters, a study found that children and adolescents who have lower CRP also seem to have lower BMI and leptin, in accordance with our results [64].

Even when categorizing the participants into LA and HA groups, both demonstrated enhancements in the quality of their diets, evidenced in the KIDMED index scores. Indeed, the LA group started the intervention with the need to improve the quality of their diet, whereas the HA group already exhibited an optimal quality of the MD. Upon completion of the intervention, both groups had a total KIDMED score over 8 points, with the HA group scoring almost 10 points. All this means that both groups concluded the study with a good adherence to the MD, in concordance with a study performed in schools with children with obesity [65]. However, interestingly, one could speculate that participants that started the intervention with a lower quality of diet had greater potential from improvement, and could approach it better and respond better to it. This was observed in a study from Brazil, which found that the group that had a lower diet quality at baseline had better health outcomes [66]. In contrast, studies performed in adults indicated that participants who showed poorer habits at baseline encountered more difficulties in adhering to the MD [67,68]. This can also be seen in our study, as although both groups achieved an optimal MD adherence, the HA group finished with a better KIDMED index score. One of the biggest achievements of this intervention can be associated with the augmentation of vegetable consumption. A total of 42 out of 44 participants incorporated regular vegetables into their diets. This can be attributed to the ready-to-eat dishes and recipes that were part of the ALINFA intervention menu, which were based on vegetables, thereby facilitating their consumption at every meal. Thirty participants finished the intervention consuming two servings of fruit daily, which puts them on the path to fulfil the nutritional recommendations [20]. Although the HA group did not attain significance regarding the change in their fruit and vegetable intake, this might be due to the smaller sample size. School-based interventions performed in some countries in healthy children also observed an increase in vegetable intake [69,70,71]. Furthermore, both groups increased consumption of grains and nuts, in line with a study in Spanish preschoolers [72]. Nut intake has been associated with enhanced educational performance in adolescents [73], suggesting that following the MD diet could have an impact on academic prospects. Furthermore, the ALADINO study found that the foods most consumed by Spanish children for breakfast were milk, chocolate, biscuits, bread, sugary cereals, and bakery products [7], and stressed the need to enhance the nutritional profile of this meal [74]. The ALINFA participants reduced the consumption of pastries and UPF for breakfast, as observed in other interventions [75,76]. Consuming a healthy and nutritional adequate breakfast has been associated with better nutrient coverage and intake throughout the remainder of the day [74]. All these aforementioned changes underline the impact a nutritional intervention can exert on diet quality. 

Although the quality of the diet improved, the eating behaviors did not change. This highlights the difficulty of changing eating behaviors in children, where parents play an essential role [77,78]. The duration of the intervention is a critical point, and 8 weeks may have not been sufficient to make real changes in behaviors, as up to 9 months could be needed to change a habit [79]. However, although it did not reach significance, a trend in decreasing the speed of eating could be seen in the LA group. Eating quickly has been associated with higher weight, adiposity, and energy intake [80,81,82]. The lack of a change in the eating pace of either group could explain why weight did not differ. 

With respect to the regressions conducted, the initial quality of diet was a predictor of adherence to the intervention, suggesting that participants with a better initial quality of their diet exhibit greater adherence to the ALINFA intervention. To our knowledge, there is no research in children that assesses the quality of diet as a determinant of adhesion to a nutritional intervention. In the adult population, some studies suggest the influence of socioeconomic status, physical activity, family support, session attendance, sleep duration, and eating schedules on compliance with an intervention [83,84,85,86]. Higher family income is positively related to consumption of fruits, vegetables, and dairy products [87]. Children whose pre-intervention diet included these foods seem to demonstrate a better adherence to the diet, in line with another study where the consumption of fruits and vegetables was positively associated with quality of diet and quality of life [88]. On the other hand, consuming sweets or candies more than once per day prior to the intervention seems to be a predictor of diminished adherence to the intervention. Indeed, UPF consumption is associated with a lower MD adherence in children [89,90].

In this context, personalized nutritional interventions, nutritional education, or dietary programs need to be conducted for children whose habits and quality of diet must be improved. Although the intervention was globally beneficial for both groups, the LA group initially had worse dietary habits. Although the margin of improvement for this group was higher, the HA group finished with better quality of diet, suggesting that participants with better habits prior to the intervention will benefit more from it. For this reason, special attention must be drawn to the LA group, as the energy and nutrient intake was lower, as well as the adherence to the MD. More emphasis needs to be placed on children showing poorer dietary habits, with the need to identify and design specific strategies for them to observe extra benefits.

The study presents some limitations. Firstly, the sample size was small. Only 20 participants were allocated to one of the groups, and only 13 participants in the HA group agreed to provide blood samples. Future studies with larger sample sizes are needed with which to compare our results. In addition, we did not have the opportunity to follow the participants for more than 8 weeks to see if the intervention was effective in the long term. Another limitation is the disparity of age in our participants, which spanned from 6 to 12, when pubertal transitions tend to happen. For this reason, two different diets were planned based on the amount of food and, therefore, the recommended intake. In addition, the socioeconomic status or the parental education could influence the adherence to the intervention [91]; however, we did not collect this information. Understanding how parents view the importance of healthy eating habits is vital, given their influence in shaping their children’s dietary choices during the stage of development. Given the absence of such research, it is a point for future studies. However, the current study also has notable strengths that could be pointed out. It is among the pioneer analyses that examines the adherence to an intervention in children. This is necessary to identify factors for optimizing future nutritional interventions in children. With food records, the adherence to the intervention can be measured explicitly. This was also a longitudinal study, which facilitated researchers to look for temporal differences. In addition, it was one of the first studies in Spain performed in healthy children that gave out free foods, thereby helping with the compliance with the study. Only three visits were scheduled, ensuring the accessibility for all participants. Moreover, the assessment encompassed not only dietary intake, but also anthropometric and biochemical parameters, thereby enriching the investigation.

## 5. Conclusions

The ALINFA program is beneficial for all participants, independent of their adherence to the intervention. Both groups improved in terms of anthropometry and body composition, as well as biochemical markers, but more benefits were identified in participants that had high adherence to the intervention. The adherence to the Mediterranean Diet improved, with increased consumption of fruits, vegetables, grains, and nuts, and a decreased intake of pastries. Baseline diet quality influences the response to the nutritional intervention. Indeed, children who have already established good dietary habits respond better, and improve more, than those who begin with poorer eating habits, even if the latter have greater margins for improvement. Special emphasis needs to be placed on participants whose initial diet is worse. Providing nutrition education or intervention programs for them should be a target.

## Figures and Tables

**Table 1:** Percentage of energy and macronutrients consumed with regards to ALINFA diet.

	Mean 8 Weeks	Week 1–2	Week 3–4	Week 5–6	Week 7–8	*p* Value
LA (n = 24)						
Kilocalories (%)	67.32 ± 7.71	69.26 ± 8.96	66.94 ± 12.49	67.68 ± 7.79	65.40 ± 10.94	0.381
Carbohydrates (%)	67.65 ± 7.19	69.77 ± 7.57	68.10 ± 12.57	67.39 ± 8.13	65.30 ± 11.16	0.298
Protein (%)	68.57 ± 8.61	70.28 ± 10.18	68.16 ± 12.55	68.76 ± 8.34	67.07 ± 11.88	0.533
Fat (%)	66.20 ± 10.20	68.16 ± 11.69	64.83 ± 14.09	66.52 ± 10.99	65.28 ± 13.04	0.521
HA (n = 20)						
Kilocalories (%)	85.02 ± 7.98	85.40 ± 9.07	82.98 ± 10.99	85.70 ± 8.28	85.98 ± 8.18	0.268
Carbohydrates (%)	84.87 ± 9.33	85.17 ± 10.99	82.94 ± 12.25	85.38 ± 9.73	86.00 ± 9.24	0.383
Protein (%)	86.51 ± 6.99	87.15 ± 8.26	85.06 ± 9.72	86.70 ± 7.86	87.15 ± 7.84	0.584
Fat (%)	83.89 ± 7.71 ^a,b^	84.70 ± 8.43 ^a,b^	80.08 ± 12.76 ^a^	85.41 ± 7.78 ^b^	85.37 ± 8.08 ^a,b^	0.029 ^a,b^

HA or LA was established according to the consumption of 75% of the prescribed diet (measured by energy intake). Values are expressed as mean ± standard deviation. a *p* values were analyzed with repeated measures ANOVA. Values with different symbols ^a,b^ are significantly different, *p* < 0.05, by Tukey post hoc test. LA = low adherence; HA = high adherence.

**Table 2:** Baseline differences and changes in anthropometry, biochemistry, and questionnaire score after the intervention depending on adherence group.

	LA (n = 24)			HA (n = 20)			Baseline Differences *p* Value ^c^	Change between Groups *p* Value ^d^
	Basal	Post- Intervention	*p* Value ^a^	Basal	Post- Intervention	*p* Value ^b^		
Anthropometry								
Weight (kg)	33.21 ± 7.63	33.03 ± 7.65	0.355	37.38 ± 12.17	36.91 ± 11.59	0.140	0.174	0.423
Height (m)	1.38 ± 0.10	1.39 ± 0.10	<0.001	1.40 ± 0.13	1.41 ± 0.14	<0.001	0.489	0.877
BMI (kg/m^2^)	17.05 (3.05)	16.62 (3.48)	0.002	17.50 (6.95)	16.82 (5.09)	0.019	0.556	0.617
BMI z-score	−0.20 (0.82)	−0.23 (0.71)	<0.001	−0.28 (1.98)	−0.39 (1.59)	0.021	0.832	0.737
Waist (cm)	58.16 (5.65)	58.50 (8.80)	0.219	60.87 (11.67)	58.30 (9.19)	0.089	0.211	0.416
SBP (mmHg)	102.00 (9.50)	105.25 (10.50)	0.159	101.50 (14.00)	100.50 (17.00)	0.360	0.433	0.870
DBP (mmHg)	68.22 ± 10.08	70.63 ± 9.98	0.036	65.95 (11.18)	64.66 (9.39)	0.966	0.493	0.254
Body composition								
Fat mass (kg)	6.09 (4.00)	6.65 (4.10)	0.528	7.10 (9.00)	6.45 (6.50)	0.002	0.671	0.030
Lean mass (kg)	25.25 (9.40)	25.15 (8.40)	0.647	27.75 (8.45)	27.20 (9.15)	0.104	0.322	0.487
Muscular mass (kg)	23.95 (8.90)	23.95 (8.05)	0.616	26.15 (8.00)	25.60 (8.60)	0.096	0.316	0.517
Total water (kg)	19.65 (7.15)	18.45 (6.50)	0.440	20.35 (6.20)	19.80 (6.65)	0.107	0.571	0.732
Biochemistry								
Glucose (mg/dL)	91.81 ± 6.25	90.22 ± 10.79	0.529	93.64 ± 6.94	92.38 ± 6.71	0.597	0.441	0.929
Insulin (µIU/mL)	9.71 ± 4.95	14.18 ± 17.07	0.249	11.01 ± 5.18	10.87 ± 5.55	0.883	0.479	0.744
HOMA-IR	1.61 (2.03)	1.58 (2.12)	0.829	1.62 (2.94)	1.26 (2.83)	0.818	0.784	0.766
TC (mg/dL)	172.89 ± 28.69	170.32 ± 27.33	0.583	174.53 ± 24.98	167.77 ± 17.12	0.142	0.868	0.532
HDL-c (mg/dL)	63.46 ± 8.11	65.10 ± 8.27	0.113	60.77 ± 11.29	60.31 ± 11.95	0.770	0.439	0.236
LDL-c (mg/dL)	98.17 ± 24.69	91.91 ± 22.98	0.144	104.01 ± 25.47	96.51 ± 22.84	0.036	0.522	0.827
TNF-α (pg/mL)	5.06 ± 1.09	4.96 ± 1.83	0.795	5.37 ± 1.67	5.26 ± 1.54	0.723	0.531	0.981
Leptin (ng/mL)	0.82 (1.53)	0.72 (0.70)	0.046	1.43 (3.55)	0.96 (1.59)	0.055	0.258	0.618
CRP (mg/dL)	0.67 (0.58)	0.32 (0.72)	0.744	0.54 (2.10)	0.36 (1.55)	0.023	0.808	0.075
Questionnaires								
KINDL	87.50 (9.72)	91.67 (8.33)	0.105	91.67 (8.33)	90.28 (8.33)	0.639	0.227	0.352
PAQ-C	2.98 ± 0.60	3.20 ± 0.47	0.120	3.25 ± 0.59	3.22 ± 0.58	0.830	0.140	0.210
KIDMED	6.13 ± 1.90	8.62 ± 2.22	<0.001	8.15 ± 2.03	9.80 ± 1.51	<0.001	0.001	0.054

Values are expressed as mean ± standard deviation, if normally distributed, or median (interquartile range), if not normally distributed. Significance considered when *p* < 0.05. ^a,b^ analyses based on Student’s *t*-test or Wilcoxon signed-rank test. ^c,d^ analyses based on Student’s *t*-test or Wilcoxon rank-sum (Mann–Whitney) test. LA = low adherence; HA = high adherence BMI = body mass index; SBP = systolic blood pressure; DBP = diastolic blood pressure; HOMA-IR = homeostatic model assessment insulin resistance; TC = total cholesterol; HDL-c = high-density lipoprotein cholesterol; LDL-c = low-density lipoprotein cholesterol; TNF-α = tumor necrosis factor-alpha; CRP = C-reactive protein; KINDL = quality of life questionnaire in children and adolescents; PAQ-C = Physical Activity Questionnaire for Children; KIDMED = Mediterranean Diet Quality Index for children and adolescents.

**Table 3:** Changes in KIDMED questionnaire by groups.

		LA (n = 24)			HA (n = 20)			
	Basal	Post-Intervention	*p* Value ^a^	Basal	Post- Intervention	*p* Value ^b^	Baseline Differences *p* Value ^c^	Change between Groups *p* Value ^d^
Takes a fruit or fruit juice every day (+1)	70.83%	87.50%	0.103	90.00%	95.00%	0.317	0.117	0.128
Has a second fruit every day (+1)	29.17%	54.17%	0.014	60.00%	85.00%	0.083	0.040	1.000
Has fresh or cooked vegetables regularly once a day (+1)	75.00%	95.83%	0.025	80.00%	95.00%	0.317	0.694	0.945
Has fresh or cooked vegetables more than once a day (+1)	33.33%	58.33%	0.014	55.00%	75.00%	0.564	0.694	1.000
Consumes fish regularly (at least 2–3/week) (+1)	54.17%	79.17%	0.058	75.00%	95.00%	0.157	0.149	0.323
Goes >1/week to a fast-food restaurant (hamburger) (−1)	16.67%	4.35%	0.083	15.00%	0%	0.083	0.153	1.000
Likes pulses and eats them >1/week (+1)	75.00%	91.67%	0.157	90.00%	100%	0.157	0.880	0.199
Consumes pasta or rice almost every day (5+/week) (+1)	4.17%	8.33%	0.564	10.00%	5.00%	0.564	0.199	0.662
Has cereals or grains (bread, pasta, etc) for breakfast (+1)	70.83%	91.67%	0.025	80.00%	95.00%	0.045	0.455	0.946
Consumes nuts regularly (at least 2–3/week) (+1)	33.33%	62.50%	0.019	50.00%	85.00%	0.014	0.484	0.908
Uses olive oil at home (+1)	100%	100%	1.000	100%	100%	1.000	0.263	1.000
Skips breakfast (−1)	4.17%	4.17%	1.000	5.00%	5.00%	1.000	0.895	0.356
Has a dairy product for breakfast (yogurt, milk…) (+1)	95.83%	91.67%	0.564	100%	95.00%	0.317	0.356	0.356
Has commercially pastries for breakfast (−1)	41.67%	12.50%	0.020	45.00%	0%	0.008	0.824	0.356
Takes two yoghurts and/or some cheese (40 g) daily (+1)	50.00%	58.33%	0.489	55.00%	75.00%	0.103	0.741	0.743
Takes sweets and candy several times every day (−1)	4.17%	4.17%	1.000	0%	0%	1.000	0.356	0.356

LA = low adherence; HA = high adherence. Data represent the percentage of children in each group who answered affirmatively to each of the items of the questionnaire. ^a,b^ *p* values are based on McNemar test. ^c,d^ *p* values are based on chi-square test. Significance considered when *p* < 0.05.

**Table 4:** Changes in scores from different eating behaviours by groups.

CEBQ Scales	LA (n = 24)			HA (n = 20)			Baseline Differences *p* Value ^c^	Change between Groups *p* Value ^d^
	Basal	Post- Intervention	*p* Value ^a^	Basal	Post- Intervention	*p* Value ^b^		
Enjoyment of food	3.17 ± 0.98	3.05 ± 0.90	0.273	3.53 ± 0.73	3.64 ± 0.78	0.144	0.185	0.058
Food responsiveness	1.80 (1.50)	2.00 (1.18)	0.164	2.10 (0.90)	2.00 (1.40)	0.717	0.321	0.788
Emotional overeating	1.75 (1.00)	2.00 (0.88)	0.205	1.88 (1.00)	2.00 (0.75)	0.499	0.404	0.178
Desire to drink	2.33 (1.34)	2.00 (0.83)	0.413	1.67 (1.00)	1.67 (1.00)	0.195	0.058	0.970
Satiety responsiveness	2.98 ± 0.72	2.92 ± 0.55	0.542	2.60 ± 0.49	2.49 ± 0.65	0.336	0.054	0.617
Slowness in eating	2.69 ± 0.88	2.82 ± 0.79	0.090	2.16 ± 0.76	2.17 ± 0.78	1.000	0.042	0.324
Emotional undereating	2.43 ± 0.72	2.46 ± 0.79	0.822	1.93 ± 0.67	1.99 ± 0.67	0.696	0.023	0.872
Fussiness	2.98 ± 0.92	2.96 ± 0.85	0.880	2.57 ± 0.76	2.65 ± 0.67	0.439	0.125	0.497

CEBQ = Child Eating Behavior Questionnaire; LA = low adherence; HA = high adherence. Values are expressed as mean ± standard deviation, if normally distributed, or median (interquartile range), if not normally distributed. ^a,b^ *p* values based on paired Student’s *t*-test or Wilcoxon signed-rank test. ^c,d^ *p* values based on Student’s *t*-test or Wilcoxon rank-sum (Mann–Whitney) test. Significance was considered with *p* < 0.05.

**Table 5:** Linear regressions between the adherence to the ALINFA intervention within different variables.

	Adherence to ALINFA Diet (%)		
	β Coefficient	*p* Value	95% Conf. Interval
KIDMED total score	1.75	0.005	0.536–2.959
KIDMED 2. Has a second piece of fruit every day	7.12	0.047	0.086–14.166
KIDMED 3. Has fresh or cooked vegetables more than once a day	10.38	0.007	2.821–17.932
KIDMED 5. Consumes fish regularly (at least 2–3/week)	9.52	0.001	3.676–15.378
KIDMED 13. Has a dairy product for breakfast (yogurt, milk…)	19.55	<0.001	10.795–28.303
KIDMED 15. Takes two yoghurts and/or some cheese (40 g) daily	5.76	0.003	0.632–10.889
KIDMED 16. Takes sweets and candy several times every day	−12.50	<0.001	−20.696–−4.305

KIDMED = Mediterranean Diet Quality Index for children and adolescents. Significance considered when *p* < 0.05.

## Data Availability

All data and material are available upon reasonable request to the corresponding author.

## Supplementary Materials

The following supporting information can be downloaded at: https://www.mdpi.com/article/10.3390/children11050559/s1, Table S1: ALINFA diet; Table S2: Nutritional information regarding ALINFA diet; Table S3: Food products, ready to eat dishes and recipes.
