# Supplementary material for: A Higher Adherence to the ALINFA Nutritional Intervention Is Effective for Improving Dietary Patterns in Children
Source: Children (Basel). 2024 May 7;11(5):559. doi: 10.3390/children11050559 (PMC11120244; doi:10.3390/children11050559)
Supplement: Supplementary file 1 [file children-11-00559-s001.zip › children-2980742-supplementary.pdf]

SUPPLEMENTARY MATERIAL

Table S1: ALINFA DIET

Week 1	DAY 1	DAY 2	DAY 3	DAY 4	DAY 5	DAY 6	DAY 7
BREAKFAST	1 glass of whole milk (200 ml) with 1 tablespoon of soluble cocoa (10 g)<br>Breakfast cereals (30g)<br>Orange (150g)	1 glass of whole milk (200 ml) with 1 tablespoon of soluble cocoa (10 g)<br>1 whole wheat toast (30 g)<br>Banana (80g)	1 glass of whole milk (200 ml) with 1 tablespoon of soluble cocoa (10 g)<br>Breakfast cereals (30g)<br>Apple (150g)	1 glass of whole milk (200 ml) with 1 tablespoon of soluble cocoa (10 g)<br>1 whole wheat toast (30 g)<br>Tangerine (150g)	1 glass of whole milk (200 ml) with 1 tablespoon of soluble cocoa (10 g)<br>Breakfast cereals (30g)<br>Kiwi (150g)	1 glass of whole milk (200 ml) with 1 tablespoon of soluble cocoa (10 g)<br>1 whole wheat toast (30 g)<br>Orange (150g)	1 glass of whole milk (200 ml) with 1 tablespoon of soluble cocoa (10 g)<br>Breakfast cereals (30g)<br>Pear (150g)
MID-MORNING SNACK	Snack: Rice, corn and peas with olive oil (1 portion)<br>1 handful of pistachios (15 g)	Tangerine (150g)<br>1 handful of walnuts (15 g)	1 small sandwich of whole wheat bread (45 g) with ham (40 g)	Banana (80g)<br>1 handful of hazelnuts (15 g)	1 small sandwich of whole wheat bread (45 g) with ham (20 g) and sliced cheese (20 g)	Snack: Rice, corn and fruit with olive oil (1 portion)<br>Tangerine (150g)	1 banana (80g)<br>1 handful of almonds (15 g)
LUNCH	Green beans with potato (1 portion)<br>Lentils with carrot (1 portion)<br>Apple (150g)	Lettuce, corn and ham salad (1 portion)<br>Asparagus and turkey burrito (1 portion)<br>1 natural yogurt with fibre topping (10 g)	Chard with serrano ham (1 portion)<br>Cod with zucchini vichyssoise (1 portion)<br>Orange (150g)	Zucchini spaghetti (1 portion)<br>Black bean burger (1 portion)<br>1 natural yogurt with red fruit topping (10 g)	Macaroni with vegetables (1 portion)<br>Gratinéed hake in dill sauce (1 portion)<br>Pear (150g)	Steamed artichokes (1 portion)<br>Borage pancakes with mushrooms (1 portion)<br>1 natural yogurt with fruit topping (10 g)	Oatmeal paella (1 portion)<br>Flexynuggets (1 portion)<br>Orange (150g)
AFTERNOON SNACK	1 natural yogurt with red fruit topping (10 g)<br>Kiwi (150g)	1 small sandwich of whole wheat bread (45 g) and sliced cheese (40 g)	1 natural yogurt with fruit topping (10 g)<br>Banana (80g)	1 small sandwich of whole wheat bread (45 g) with serrano ham (40 g)	1 natural yogurt with fibre topping (10 g)<br>Strawberries (150g)	1 small toast of whole wheat bread (45 g) with tomato and fresh cheese (60 g)	1 toast of whole wheat bread (45 g) with cottage cheese Nutella ,

							cocoa and nuts (1 portion)
DINNER	Vegetable flan with cheese and oregano with tomato garnish (1 portion)<br>1 natural yogurt with fibre topping (10 g)	Battered hake in oatmeal popcorn and zucchini cream (1 portion)<br>Pear (150g)	Chicken wok with vegetables, curry and white rice (1 portion)<br>1 natural yogurt with fibre topping (10 g)	Baked dumplings (1 portion)<br>Apple (150g)	Cabbage omelette with peas (1 portion)<br>1 natural yogurt with red fruit topping (10 g)	Grilled pork tenderloin with french fries (1 portion)<br>Pear (150g)	Chickpea meatballs with tuna and tomato (1 portion)<br>1 natural yogurt with red fruit topping (10 g)

Week 2	DAY 1	DAY 2	DAY 3	DAY 4	DAY 5	DAY 6	DAY 7
**BREAKFAST**	1 glass of whole milk (200 ml) with 1 tablespoon of soluble cocoa (10 g)<br>1 whole wheat toast (30 g)<br>Kiwi (150g)	1 glass of whole milk (200 ml) with 1 tablespoon of soluble cocoa (10 g)<br>Breakfast cereals (30g)<br>1 banana (80g)	1 glass of whole milk (200 ml) with 1 tablespoon of soluble cocoa (10 g)<br>1 whole wheat toast (30 g)<br>Pear (150g)	1 glass of whole milk (200 ml) with 1 tablespoon of soluble cocoa (10 g)<br>Breakfast cereals (30g)<br>Apple (150g)	1 glass of whole milk (200 ml) with 1 tablespoon of soluble cocoa (10 g)<br>1 whole wheat toast (30 g)<br>Orange (150g)	1 glass of whole milk (200 ml) with 1 tablespoon of soluble cocoa (10 g)<br>Breakfast cereals (30g)<br>Kiwi (150g)	1 glass of whole milk (200 ml) with 1 tablespoon of soluble cocoa (10 g)<br>1 whole wheat toast (30 g)<br>Tangerine (150g)
**MID-MORNING SNACK**	Snack: Rice, corn and peas with olive oil (1 portion)<br>Tangerine (150g)	1 small sandwich of whole wheat bread (45 g) with ham (40 g)	1 small sandwich of whole wheat bread (45 g) with serrano ham (40 g)	Tangerine (150g)<br>1 handful of cashews (15 g)	Snack: Rice, corn and fruits with olive oil (1 portion)<br>Apple (150g)	Banana (80g)<br>1 small sandwich of whole wheat bread (30 g) and ham (20 g)	Apple (150g)<br>1 small sandwich of whole wheat bread (30 g) and ham (20 g)
**LUNCH**	Lettuce salad, carrot strips and green olives (1 portion)<br>Chickpeas, curry and spinach (1 portion)<br>1 natural yogurt with fibre topping (10 g)	Gratinéed cauliflower (1 portion)<br>Battered pomfret in tomato sauce (1 portion)<br>Apple (150g)	Tomato and fresh cheese salad (1 portion)<br>Lentils with pumpkin and chicken (1 portion)<br>1 natural yogurt with red fruit topping (10 g)	Milanese rice (1 portion)<br>Vegetable omelette (1 portion)<br>Pear (150g)	Vegetable stew (1 portion)<br>Flexiballs (1 portion)<br>1 natural yogurt with fibre topping (10 g)	Spaghetti with tomato sauce and vegetables (1 portion)<br>Baked cod with lemon (1 portion)<br>Tangerine (150g)	Zucchini cream (1 portion)<br>Flexiburger (1 portion)<br>1 natural yogurt with red fruit topping (10 g)
**AFTERNOON SNACK**	1 small sandwich of whole wheat bread (45 g) with serrano ham (40 g)	1 natural yogurt with fruit topping (10 g)<br>Strawberries (150g)	Apple (150g)<br>1 handful of walnuts (15 g)	1 small sandwich of whole wheat bread (45 g) with sliced cheese (40 g)	1 handful of hazelnuts (15 g)<br>Tangerine (150g)	1 small toast of whole wheat bread (45 g) with tomato and goat cheese (40 g)	1 glass of whole milk (150 ml)<br>Beet brownie (1 portion)
**DINNER**	Green bean, smoked salmon, sesame, walnut and fruit salad (1 portion)	Pork loin in apple sauce with white rice (1 portion)	Vegetables with pasta (1 portion)<br>Orange (150g)	Chicken schnitzel with baby carrots (1 portion)	Baked sweet potato stuffed with egg and melted cheese (1 portion)	Ham omelette with tomato sauce and vegetables (1 portion)	Oatmeal and pumpkin broth rice (1 portion)<br>Banana (80g)

	Pear (150g)	1 natural yogurt with fibre topping (10 g)		1 natural yogurt with fruit topping (10 g)	Banana (80g)	1 natural yogurt with fruit topping (10 g)	

* Daily 1 portion of whole wheat bread (30 g) at lunch and dinner.

Company products

Ready-to-eat dishes

Recipes to make at home

Table S2: NUTRITIONAL INFORMATION OF ALINFA DIET

	AMDR	Younger age group	Older age group
Carbohydrates	**45-65%**	205.00 g **(48.5%)**	227 g **(49%)**
Protein	**10-30%**	83.00 g **(19.5%)**	91.00 g **(19%)**
Lipids	**25-35%**	60.00 g **(32%)**	66.00 g **(32%)**
SFA		19.40 g **(10%)**	21.15 g **(10%)**
MUFA		28.75 g **(15%)**	33.30 g **(16%)**
PUFA		11.40 g **(6%)**	13.10 g **(6%)**
Fibre		31.50 g	33.50 g
Sugar		87.90 g	91.10 g
Iron		12.35 mg	13.70 mg
Calcium		912.35 mg	967.00 mg
Sodium		2542.90 mg	2785.30 mg
Vitamin D		3.90 µg	4.20 µg

Note: AMDR=Acceptable Macronutrient Distribution Ranges; SFA= saturated fatty acids; MUFA= monounsaturated fatty acids; PUFA= polyunsaturated fatty acids.

TABLE S3: FOOD PRODUCTS, READY-TO-EAT DISHES AND RECIPES

**Industry**	**Dish**	**Ingredients**
**GRUPO APEX S.A.**	▪ Snack 1	*Rice, corn and peas flour, olive oil*
	▪ Snack 2	*Rice and corn flour, banana, orange and hawthorn dehydrated berry, olive oil*
**Harivenasa S.L**	▪ Red fruit topping	*Oat flakes, pumpkin seeds, blueberries, goji berries, cherries, strawberries*
	▪ Fibre topping	*Oat flakes, pumpkin seeds, sunflower seeds, golden linseeds, brown linseeds, kiwi*

	▪ Fruit topping	*Oat flakes, sunflower seeds, pumpkin seeds, blueberries, pineapple, apple, goji berries, cherries*
**Alimentos Sanygran S.L**	▪ Flexinuggets	*Chicken, hydrated vegetable extruded (soy, rice and bean flours), salt, spices, tomato and beet powder*
	▪ Flexiballs	*Hydrated vegetable extruded (soy, rice and bean flour), beef, soy protein, salt, soy sauce, spices, beet powder, wheat flour, sunflower oil*
	▪ Flexiburgers	*Hydrated vegetable extruded (soy, rice and bean flour), beef, soy protein, salt, soy sauce, onion, garlic, beet powder*
**Industrias Alimentarias de Navarra S.A.U**	▪ Oatmeal paella	*Oat, braised chicken breast, pea, sweet corn, green bean, soybeans, olive oil, red bean, sunflower seeds, onion, salt, saffron extract, soy lecithin*
	▪ Vegetable stew	*Green beans, artichokes, carrot, mushroom, olive oil, salt*
	▪ Vegetables with pasta	*Pasta, tomato, green bean, zucchini, onion, olive oil, green and red pepper, orange rind, sugar, salt*
**Irigoyen Comedor Saludable S.L.**	▪ Green beans with potato	*Green beans, potato, olive oil, garlic, salt*
	▪ Lentils with carrot	*Lentils, tomato, onion, carrot, red and green pepper, olive oil, garlic, salt*
	▪ Vegetable flan	*Pasteurized egg, zucchini, potato, emmental cheese, carrot, red and green pepper, onion, tomato, olive oil, salt, oregano*
	▪ Chard with serrano ham	*Chard, serrano ham, olive oil, garlic, salt*
	▪ Cod with zucchini vichyssoise	*Cod, potato, zucchini, breadcrumbs, butter, olive oil, salt*
	▪ Chicken wok with vegetables	*Chicken, red and green pepper, carrot, onion, potato, vinegar, olive oil, salt*
	▪ Macaroni with vegetables	*Pasta, broccoli, pumpkin, red/green pepper, olive oil, salt*

**National Center for Technology and Food Safety**	▪ Gratinated hake in dill sauce	*Hake, whole milk, onion, leek, yolk, corn flour, olive oil, dill, salt*
	▪ Cabbage omelette with peas	*Cabbage, pasteurized egg, potato, peas, olive oil and salt*
	▪ Gratineed cauliflower	*Cauliflower, wheat flour, butter, emmental cheese and white pepper and salt*
	▪ Battered pomfret in tomato sauce	*Pomfret, tomato sauce, olive oil, wheat flour, pasteurized egg, red/green pepper, onion, garlic, salt, oregano*
	▪ Pork loin in apple sauce with rice	*Pork, onion, apple, olive oil, vinegar, ginger, salt*
	▪ Milanese rice	*Rice, carrot, mushroom, raisins, emmental cheese, olive oil, oregano, salt*
	▪ Vegetable omelette	*Pasteurized egg, red/green pepper, zucchini, carrot, olive oil, salt*
	▪ Chicken schnitzel with baby carrots	*Chicken, carrot, olive oil, garlic, salt*
	▪ Asparagus and turkey burrito	*Asparagus, turkey breast, mozzarella cheese, wheat wafers, soy sauce*
	▪ Zucchini spaghetti	*Zucchini, pistachios, garlic, tomato, parmesan cheese, olive oil, salt*
	▪ Bean, coco and beetroot burger	*Beans, beetroot, onion, mushrooms, oat flakes, coconut, pepper, parsley, olive oil, salt*
	▪ Steamed artichokes	*Artichokes, lemon, pepper, cooking cream, olive oil, salt*
	▪ Oatmeal crepes with mushrooms, borage and veal	*Veal, mushrooms, borage, onion, carrot, tomato, flour, eggs, oat milk, butter, sugar, salt*
	▪ Hake battered in oats and zucchini	*Hake, wheat flour, eggs, oat flakes, zucchini, carrots, cheese, milk, salt*
	▪ Zucchini cream	*Zucchini, potato, cheese, olive oil, salt*
	▪ Baked pasties	*Prawns, pork, garlic, carrot, egg, patty wafers, olive oil, salt*

	▪ Chickpea meatballs with tuna and tomatoes	*Tuna, chickpeas, chickpea flour, tomato, onion, carrot, garlic, parsley, sweet paprika, pepper, olive oil, salt*
	▪ Chickpeas with curry and spinach	*Chickpeas, onion, spinach, tomato, ginger, garlic, turmeric, olive oil, salt*
	▪ Lentils with pumpkin and chicken	*Lentils, chicken breast, pumpkin, onion, sweet paprika, olive oil, salt*
	▪ Baked sweet potato with eggs	*Sweet potato, leek, onions, mozzarella cheese, thyme, eggs, olive oil, salt*
	▪ Fake pumpkin and oatmeal rice stew	*Oat groats, onion, carrot, leek, pumpkin, celery, hake, bread, tomato, butter, parsley wine, olive oil, salt*
	▪ Ham omelette with vegetables	*Eggs, ham, tomato, onion, red and green pepper, zucchini, olive oil, salt*
	▪ Grilled pork with french fries	*Pork tenderloin, potato, olive oil, salt*
	▪ Vegetable and tomato spaghetti	*Spaghetti, onion, carrot, red pepper, zucchini, tomato sauce, olive oil, salt*
	▪ Salad 1	*Tomato, soft cheese, olive oil, salt*
	▪ Salad 2	*Lettuce, carrot, green olives, olive oil, salt.*
	▪ Salad 3	*Green beans, salmon, sesame, walnuts, orange, pita bread, olive oil, salt*
	▪ Salad 4	*Lettuce, corn, ham, olive oil, salt*
	▪ Baked cod	*Cod, lemon, olive oil, salt.*
	▪ Beetroot brownie	*Beetroot, butter, chocolate, eggs, oat flour, cocoa and baking powder, salt*
	▪ Cottage cheese, cocoa and nuts cream	*Cottage cheese, cocoa powder, almond powder, hazelnuts, honey*
